# Effect of probiotic supplementation on productive performance and epithelial intestinal integrity of broiler chickens exposed to heat stress

**DOI:** 10.1007/s11250-025-04488-3

**Published:** 2025-05-30

**Authors:** Ana Cecilia Hernández-Coronado, Miguel Cervantes, Fernanda González, Alan Valle, Nestor Arce, Nydia Vásquez, Hugo Bernal, Adriana Morales

**Affiliations:** 1 Universidad Autónoma de Baja California, Mexicali, B.C México; 2 Universidad Autónoma de Nuevo León, Monterrey, N.L México

**Keywords:** Heat stress, Chicken, Probiotic, Intestinal integrity

## Abstract

Heat stress (HS) impacts performance and intestinal homeostasis of broiler chickens. Probiotic represents an alternative to counteract those negative effects. This study evaluated the performance and intestinal integrity of HS chickens supplemented with a *B. subtilis* based probiotic in two 35-d periods. Period 1 was conducted under thermoneutral conditions (TN; 25.7 ± 1.7 °C) using 150 one-day old chickens (Ross-308) randomly assigned to two dietary treatments: TN birds fed a standard diet without (TN-S) or added with 0.05% probiotic (TN-P)*.* Period 2 was conducted under HS (29.3 ± 2.6 °C) with 120 one-day old chickens fed the standard diet without (HS-S) or with 0.05% probiotic (HS-P). On day 35, ten birds per treatment were sacrificed and jejunum was collected. HS and probiotic supplementation reduced feed intake from d-1 to d-35 (*P* < 0.01). Feed conversion from day 1 to 21 was better in HS compared to TN chickens, but the opposite occurred from d-1 to d-35 (*P* < 0.01). Overall, from d-1 to d-35, body weight and daily gain were lower in HS than TN chickens (*P* < 0.01), but these variables were higher in HS-P than HS-S chickens (*P* < 0.05). HS chickens reduced villi height, and crypt depth, and increased villi height:crypt depth ratio compared to TN chickens (*P* < 0.01), but probiotic supplementation increased them (*P* < 0.01). Probiotic supplementation increased claudin-5 expression during TN and HS periods (*P* < 0.05), and TJP-1 during TN period (*P* < 0.05), but decreased occludin expression during HS (*P* < 0.05). In general, *Bacillus subtilis* supplementation positively impacts performance and epithelium integrity of the small intestine of HS broiler chickens.

## Introduction

Climate change is modifying many ecosystems around the world, especially in tropical and subtropical countries, where negative effects on animal production are observed (Cheng et al. 2022). Within the poultry industry, consequently, great concern has been generated about production and well-being of chickens exposed to heat stress conditions (Vandana et al. 2021).

Broiler chickens are particularly sensitive to high ambient temperature (AT); above 25 °C, they are unable to maintain a balance between heat production and heat loss (Wasti et al. 2020; Kumar et al. 2021), provoking increments in body temperature (Barrett et al. 2019) and resulting in heat stress (HS). At physiological and productive levels, broilers under HS increase water consumption and respiration rate, modifies their behavior, and decrease food intake in order to reduce heat production, but these changes resulted in detrimental productivity and animal well-being (Wasti et al. 2020; Goel 2021). In addition, the digestive tract is directly affected by HS as evidenced by the reported damage and thinning of the intestinal epithelium, as well as histomorphology alterations, including rupture of intestinal villi, detachment of epithelial cells, and intestinal barrier dysfunction (Lambert 2009; He et al. 2018; Rostagno 2020). Moreover, apparent changes in the intestinal microbiota in favor of non-beneficial microorganisms may further depress the digestive tract function of birds.

Probiotics supplementation, on the other hand, seems to help regulating the intestinal microbiota composition, improve the digestibility and absorption of nutrients, regulate the cytokines expression alleviating intestinal inflammatory response, and improve intestinal morphology and barrier function of broilers (Abd El-Hack et al. 2020; Zhang et al. 2022; Yosi and Metzler-Zebeli 2023), among other benefits.

We speculate that, the intestinal barrier and epithelial integrity restoration observed when birds are supplemented with probiotics could also be observed in HS birds, providing an alternative to improve their health, intestinal function and performance. The aim of this study was to evaluate the effect of supplementing a probiotic on performance and epithelial intestinal integrity (histomorphology of jejunal epithelia and relative expression of tight junction proteins) of chickens exposed to HS conditions.

## Material and methods

### Animals and experimental procedure

All chickens in this study were cared for in accordance with the guidelines established in the Official Mexican Regulations on Animal Care (NOM-062-ZOO-1999 2001), and approved by the Ethical Committee of Institute of Agricultural Sciences at the Universidad Autónoma de Baja California, Mexico. This study was performed in Northwestern Mexico, where summer ambient temperature raises up to 45 °C.

The experiment consisted of two 35-d periods: period 1 was conducted during October-December of 2021, when AT fluctuated from 14 to 25 °C, defined as thermoneutral (TN); period 2 was conducted during April-June, when AT ranged from 23 to 32 °C, defined as HS. Both periods were conducted under natural climatic conditions such as those under practical production systems. In both periods, a total of 270 one-day old chickens (Ross-308) from a commercial farm were allocated into cages of 0.50 × 0.60 m and 1.16 × 1.11 m, with sawdust bed, and equipped with feeder and drinker. Birds were fed standard starting (d1-d20) and growing (d21-d 35) diets based on wheat, soybean meal and added with free Lys, Thr, and Met to meet the birds requirements according to NRC (1994; Table 1). During first week, birds were offered oral serum with vitamins and electrolytes and, during the whole experiment, birds had ad libitum access to feed and water. Light was turned on during 24 h a day. During the experiment, relative humidity and AT inside the room where birds were allocated, were recorded every 15 min with a higrothermograph (Thermotracker HIGRO, iButtonLink LLC, Whitewater, WI, USA).

**Table 1:** Composition of experimental diets for starting (day 1–21) and growing (day 22–35) periods

Ingredient (g kg^−1^)	**Starter** **0 a 21 d**	**Grower** **22 a 35 d**
Wheat	700	740.5
Soybean meal	225	190
Canola oil	30	30
L- Lysine.HCl	2.1	2.1
L- Threonine	1.2	1.2
DL-Methionine	2.2	1.2
Calcium carbonate	17.0	17.5
Dicalcium phosphate	15.0	10.0
Salt	3.5	3.5
Vitamin and mineral premix^a^	4.0	4.0

^a^Supplied per kg of diet: vitamin A, 4,800 IU; vitamin D3, 800 IU; vitamin E, 4.8 IU; vitamin K3, 1.6 mg; riboflavin, 4 mg; D-pantothenic acid, 7.2 mg; niacin, 16 mg; vitamin B12, 12.8 mg; Zn, 64 mg; Fe, 64 mg; Cu, 4 mg; Mn, 4 mg; I, 0.36 mg; Se, 0.13 mg

For the TN period, a total of 150 one-day old chickens were weighed and distributed into 6 cages (0.50 × 0.60 m) of 25 birds per cage, according to a randomized complete block experimental design using the initial body weight as blocking factor. Then, the cages were randomly assigned to two dietary treatments: TN birds fed the standard diet (TN-S) or birds fed the same diet, but added with 0.05% of a probiotic based on *B. subtilis* (DSM 32315) containing at least 2 × 10^9^ ufc/gr (TN-P). On day 21, birds were reallocated to cages of 1.16 × 1.11 m, 8 birds per cage, and continue fed their respective growing diet until day 35. For the HS period, a total of 120, one day-old chickens were weighed and distributed into 6 cages, 20 birds per cage, 3 cages per treatment, and fed the standard (HS-S) or the probiotic supplemented diet (0.05% of the *B. subtilis* probiotic; HS-P), until day 21. Birds density was reduced during this period to minimize further stress. Diet assignment was similar to TN conditions. On day 21, birds were moved to cages of 1.16 × 1.11 m, 8 birds per cage, and continue fed their respective diet until day 35. During both periods, chickens and feed were weighed every week, in order to estimate the feed intake, average daily gain and feed conversion rate. Mention should be made that chickens used during the HS period were 16% lighter than those employed during the TN period (36.3 *vs* 43.4 gr; *P* < 0.001). We did not have control over the initial body weight of the birds as they were obtained from a commercial farm. Nevertheless, within each period (TN-S vs. TN-P; HS-S vs. HS-P), the initial BW did not differ among dietary treatments.

### Sample collection

On day 35, ten birds per treatment were randomly selected and euthanized by cervical dislocation and exsanguination according to Official Animal Care Regulation (NOM-033-SAG/ZOO-2014 2015). Following euthanasia, the jejunum was immediately washed with sterile saline physiological solution. Then, small segments of about 0.3 cm were collected into 2.0 ml-microtubes, frozen in liquid nitrogen, and stored at −80 °C until gene expression analysis. Also 5 cm segments were collected into buffered formaldehyde 3.7% solution for gut histomorphology analysis.

### Histo-morphology of jejunum

The samples from jejunum fixed in formaldehyde buffer were embedded in paraffin blocks and cut into 3 μm slices. The sections were deparaffinizated, dehydrated, and stained with hematoxilin-eosin (Driscoll and Ryan 1978). The structure of the mucosae was observed under 10 × magnification with an optical microscope (PrimoStar; Zeiss, Germany), and microphotographs were taken with a microscope camera (VE-LX1000; Velab, Pharr,Texas, USA), resolution of 10 megapixels. For each sample, 10 well-oriented villi were measured for villi height (VH), villi width (VW), and crypt depth (CD), and the VH:CD rate was calculated. Measures for VH, VW, and CD were analyzed with the software Image J2 version 1.53 (Rueden et al. 2017). The system was calibrated by changing the pixels of the image to a known value in microns.

### Tight junction expression analysis

Frozen samples from jejunum were processed to purify RNA. Jejunal tissue was pulverized on liquid nitrogen using a mortar; 50 mg of each sample was collected into 2.0 ml microtubes containing 530 μl of Trizol Reagent (Invitrogen, Corp.). Then samples were processed according to the protocol of Direct-zol RNA Miniprep Plus Kit (Zymo Research, Orange, CA, USA). Integrity or total RNA was analyzed by 1% agarose gel electrophoresis; all samples had a 28S:18S ratio of 2.0:1.0 (Sambrook and Russell 2001). Purity was estimated by absorbance at a 260/280 ratio with a spectrophotometer (Genesys 50, Thermo Fisher Scientific, Carlsbad, CA, USA) that ranged from 1.8 to 2.0 in all RNA samples.

Approximately 2 μg of total RNA was treated with 1 U of DNase I (Thermo Scientific), before reverse transcription reactions were performed using random hexamers as we previously described (García et al. 2015). The concentration of complementary DNA (cDNA) samples was quantified into a spectrophotometer and diluted into a final concentration of 50 ng/μl.

Specific primers for tight junction protein-1 (TJP1), tight junction protein-2 (TJP2), claudin-1 (CLDN1), claudin-5 (CLDN5), occludin (OCLN), and glyceraldehyde-3-phosphate dehydrogenase (GAPDH) were designed according to their mRNA sequences published in the Genbank (Table 2). Endpoint PCR were carried out to standardize the amplification conditions for primers of every mRNA; also, products of those PCR were sequenced at GENEWIZ facilities (Azenta Life Sciences, South Plainfield, NJ, USA). Sequencing results revealed that the PCR products for TJP1, TJP2, CLDN1, CLDN5, OCLN and GAPDH were 100% homologous to their respective sequences reported in GenBank.

**Table 2:** Primers used for the qPCR analyses of messenger RNA codding for tight junction protein 1, tight junction protein 2, claudin 1, claudin 5, occluding and glyceraldehyde-3-phosphate dehydrogenase

mRNA	**Primer sequencing (5ʹ → 3ʹ)**	**Amplicon bp**
***Gallus gallus* tight junction protein 1 (TJP1), mRNA. (XM_046925214.1)**		
	Fw 5ʹTAAAGCCATTCCTGTAAGCC3ʹ	243
	Rv 5ʹGTTTCACCTTTCTCTTTGTCC3ʹ	
***Gallus gallus* tight junction protein 2 (TJP2), mRNA. (NM_001396728.1)**		
	Fw 5ʹAGGCAAATCATTGAGCAGGA3ʹ	240
	Rv 5ʹATTGATGGTGGCTGTAAAGAG3ʹ	
***Gallus gallus* claudin 1 (CLDN1), mRNA. (NM_001013611.2)**		
	Fw 5ʹGCTGATTGCTTCCAACCAG3ʹ	140
	Rv 5ʹAGGTCAAACAGAGGTACAAG3ʹ	
***Gallus gallus* claudin 5 (CLDN5), mRNA. (NM_204201.2)**		
	Fw 5ʹCATCACTTCTCCTTCGTCAG3ʹ	111
	Rv 5ʹGCACAAAGATCTCCCAGGTC3ʹ	
***Gallus gallus* occludin (OCLN), mRNA. (NM_205128.1)**		
	Fw 5ʹTCATCGCCTCCATCGTCTAC3ʹ	240
	Rv 5ʹTCTTACTGCGCGTCTTCTGG3ʹ	
*Gallus gallus* glyceraldehyde-3-phosphate dehydrogenase (GAPDH), mRNA. (NM_204305.2)		
	Fw 5ʹGCTGAATGGGAAGCTTACTG3ʹ	216
	Rv 5ʹAAGGTGGAGGAATGGCTG3ʹ	

The expression of mRNA for the five tight junction proteins was analyzed by quantitative PCR (qPCR) assays, and GAPDH was used as endogenous control to normalize variations in mRNA. Reactions for qPCR were prepared using the commercial kit SYBR Green/ROX qPCR Master Mix (Thermo Fisher Scientific), reactions contained 50 ng of cDNA, 0.5 μM of each primer, 12.5 μl of 2 × SYBR green Master Mix, and nuclease free water to complete a final volume of 25 μl. Samples for every specific tight junction protein and endogenous control were analyzed by duplicate, also three-duplicate negative controls were amplified at the same time (qPCR reactions without DNA template; qPCR reactions without primers; and qPCR reactions without SYBR Mix). The qPCR amplifications were carried on the C1000 Touch Thermal Cycler equipped with the CFX96 Real Time System (BioRad, Herefordshire, England). For the qPCR amplifications, this equipment was programed as follows: initial denaturing period of 5 min at 95 °C; 40 cycles of amplification (denaturing, 15 s at 95 °C; annealing, 15 s at 58 °C; extension 30 s at 72 °C), and melting curve from 60 to 90 °C; fluorescence was measured at the end of every cycle and every 0.5 °C during the melting. Results of mRNA expression for each tight junction protein were analyzed according to the comparative Ct method described by Livak and Schmittgen (2001), and expressed as 2^ΔΔCt^, normalized by the GAPDH expression in every sample.

### Statistical analysis

The results of productive performance, gene expression and intestinal histomophology were analyzed according to a randomized complete block design, with a 2 × 2 factorial arrangement, using the software Statistix 10.0. The effects of ambient temperature (AT; TN and HS) and probiotic supplementation (Pro), as well as their interaction were analyzed. However, because the initial BW of chickens differed significantly among periods, data from each period were also analyzed separately, and pair-wise comparison were made (Tukey´s test). Individual bird was considered as the experimental unit. Probability levels of *P* ≤ 0.05 and 0.05 < *P* ≤ 0.10 were defined as significant differences and tendencies, respectively.

## Results

**Fig. 1:**
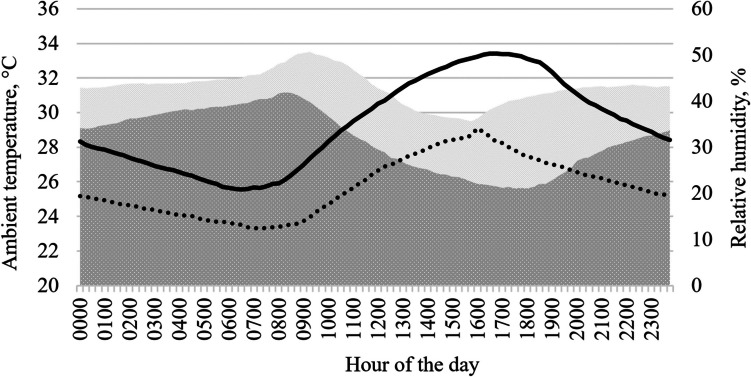
Ambient temperature and relative humidity recorded inside the room during both heat stress and thermoneutral periods. Lines indicate ambient temperature (dotted line for TN and solid line for HS). Shadows correspond to relative humidity (clear gray for TN and dark gray for HS)

The average ambient temperature (AT) and relative humidity during the TN period were 25.7 ± 1.7 °C and 43.1 ± 3.6%, respectively. In contrast, during the HS period, the AT was 29.3 ± 2.6 °C and relative humidity was 37.7 ± 6.5% (Fig. 1). The average AT recorded during the first 21 d of HS and TN periods were 29.0 ± 2.9, and 27.3 ± 2.3 °C, respectively; and from day 22 to 35, AT during HS and TN periods were 29.9 ± 3.8 and 23.7 ± 2.5 °C, respectively.

Performance of chickens is presented in Table 3. The interaction AT x Pro in BW and daily weight gain at 21 and 35 days was significant (*P* < 0.05). Regardless of the lower initial BW, the average BW and ADG of HS chickens at day 21 was higher than that of TN chickens but, at day 35, TN chickens were heavier and gained more weight per day than HS birds (*P* < 0.01). When analyzed separately by period, there was no effect of supplementing the probiotic on BW at 21 and 35 days during the TN period, but it was higher in the probiotic supplemented chickens during the HS period. The ADG of the TN chickens was not affected by the probiotic from day 1 to 21, but it was lower in the TN-P chickens from day 21 to 35 (*P* < 0.05). In contrast, during the HS period, the ADG of the HS-P chickens from day 1 to 21 was higher (*P* < 0.05) but there was no difference during the 21 to 35 day phase (*P* > 0.10). Average daily feed intake was not affected neither by AT or Pro supplementation from day 1 to 21 (*P* > 0.10), however there was a significant effect of both AT and the probiotic from day 21 to 35 (*P* < 0.01). Feed conversion rate from day 1 to 21 was better in HS compared to TN chickens (*P* < 0.05), but it was the opposite from day 21 to 35 (*P* < 0.01).

**Table 3:** Productive performance of chickens under thermoneutral conditions fed a standard diet (TN-S) or the probiotic supplemented diet (TN-P); or heat stress conditions fed a standard diet (HS-S) or the probiotic supplemented diet (HS-P)

	Treatment					Contrasts^a^, *P value*		
Parameter	TN-S	TN-P	HS-S	HS-P	SEM	AT	Pro	AT x Pro
**Body weight, g**								
Day 1	43.3	43.4	36.1	36.4	0.4			
Day 21 *	620.4	606	630.2x	684.6y	14	< 0.01	ns	< 0.05
Day 35 *	2064	1974	1542x	1609y	35	< 0.01	ns	< 0.05
**Average daily gain, g**								
Day 1 to 21 *	27.5	26.8	28.3x	30.9y	2.6	< 0.01	ns	< 0.05
Day 21 to 35 ^†^	102.0a	96.4b	64.2	65.4	1.7	< 0.01	ns	< 0.05
**Average daily feed intake, g**								
Day 1 to 21	50.1	48.2	47.3	49.1	1.3	ns	ns	ns
Day 21 to 35	157.3	145.1	128.3	119.8	2.9	< 0.01	< 0.01	ns
**Feed conversion rate**								
Day 1 to 21	1.97	1.89	1.84	1.72	0.07	< 0.05	ns	ns
Day 21 to 35	1.55	1.48	2.01	1.83	0.04	< 0.01	< 0.01	ns

^a^ Contrasts: AT, effect of ambient temperature; Pro, effect of probiotic supplementation; AT x Pro: Interaction between AT and Pro supplementation

^†^ Within rows, a, b, indicates probiotic effect (*P* < 0.05) during TN conditions

* Within rows, x, y indicates probiotic effect (*P* < 0.05) during HS conditions

The intestinal histomorphology in jejunum of chickens at 35 days of age is presented in Table 4. On average, the TN chickens had larger villi height, villi width, and crypt depth, but smaller villi height:crypt depth ratio than HS chickens (*P* < 0.01), regardless of the diet they consumed. Similarly, chickens fed the diet supplemented with the probiotic had increased villi height, villi width, and crypt depth, and smaller villi height:crypt depth ratio than chickens fed the standard diet (*P* < 0.01) during both the TN and HS periods. Except for villi height:crypt depth (*P* < 0.01), there was no interaction between AT and the supplementation of probiotic.

**Table 4:** Intestinal histomorphology of chickens under thermoneutral conditions fed a standard diet (TN-S) or the probiotic supplemented diet (TN-P); or heat stress conditions fed a standard diet (HS-S) or the probiotic supplemented diet (HS-P)

Variable (µm)	Treatment					Contrasts^a^, *P* value		
	TN-S	TN-P	HS-S	HS-P	SEM	AT	Pro	AT x Pro
Villi height (VH)	1109	1197	927	1204	10	< 0.01	< 0.01	ns
Villi width (VW)	99	148	87	138	2	< 0.01	< 0.01	ns
Crypt depth (CD)	87	136	78	120	2	< 0.01	< 0.01	ns
VH:CD ratio	13.7	8.9	12.0	10.2	0.2	ns	< 0.01	< 0.01

^a^Contrasts: AT, effect of ambient temperature; Pro, effect of probiotic supplementation; AT x Pro: Interaction between AT and Pro supplementation

**Fig. 2:**
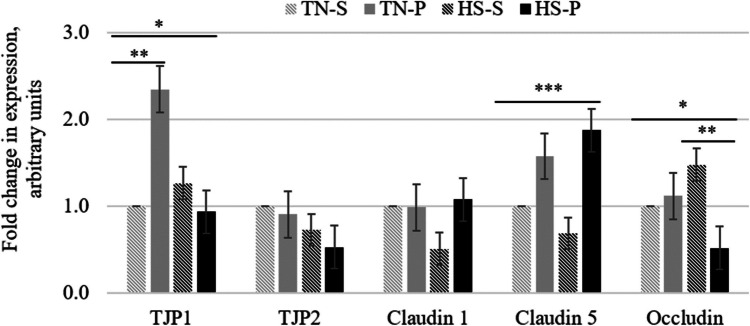
Effect of heat stress and probiotic supplementation on relative expression of mRNA codding for tight junction protein-1, tight junction protein-2, claudin-1, claudin-5, and occludin, in jejunum of chickens. Results for thermoneutral chickens fed a probiotic supplemented diet (TN-P), and chickens under heat stress conditions fed a standard diet (HS-S) or the probiotic supplemented diet (HS-P) are relative to thermoneutral chickens fed the standard diet (TN-S). * AT x Pro, *P* < 0.10; ** TN-S vs. TN-P, *P* < 0.05; HS-S vs. HS-P, *P* < 0.05; *** Pro effect (TN-C + HS-C vs. TN-P + HS-P), *P* < 0.05

The relative expression of tight junction proteins in jejunum is shown in Fig. 2. The probiotic supplementation increased the expression of claudin-5 during both the TN and HS periods (*P* < 0.05), but had no effect on TJP2 and claudin-1 expression. The interaction AT x Pro tended to be significant for TJP1 and occludin (*P* < 0.10). Accordingly, chickens fed the probiotic supplemented diet increased the expression of TJP1 during the TN period but it had no effect under HS period (HS-S and HS-P; *P* > 0.10). In contrast, the probiotic supplementation decreased the expression of occludin during the HS period, but had no effect under TN conditions.

## Discussion

Avian production is highly sensible to ambient conditions as the optimal ambient temperature for chickens ranges between 18–25 °C; under or above that range, chickens may show physiological changes that could compromise welfare, health and production (Vandana et al. 2021; Salem et al. 2022). In this study, the average AT during the TN period was slightly above birds comfort temperature, but the thermal sensation probably did not increase because the RH was under 50% (Steadman 1979). However, the AT during the HS period was above birds comfort temperature all the time (≥ 25.6 °C); moreover, AT was above 30 °C during 10 h every day (from 1100 to 2100 h). Those observations confirm that chickens were exposed to heat stress and might explain at least partially the response observed in the productive and intestinal parameters observed during the trial.

As mentioned previously, although all the birds included in the present study were from the same line and same hatchery, the average birth weight of chickens born during April, for the HS period, was 16% lighter than those born on October and used for the TN period. Nevertheless, the BW and ADG of chickens exposed to HS conditions were 7.2 and 9.1% higher at the end of the first 21 days of the study, respectively, regardless of the diet (with or without the probiotic) they consumed, as compared to the TN chickens. Young and light birds require higher AT conditions and are less sensitive to high AT than heavy birds, due to their heat necessity to maintain normal body temperature (Cahaner and Leenstra 1968; Weinert 2010; Zulkifli et al. 2018). Thus, the higher ADG of HS chickens during the first 21 days indicate a more favorable AT condition (around 1.7 °C higher) than those of TN chickens. In contrast, the ADG and BW of TN chickens from day 21 to 35 were around 35 and 22% higher, respectively, than in HS birds. As birds become heavier they also become more sensible to high AT (Sandercock et al. 2001; Gogoi et al. 2021), such as those recorded under the HS conditions of the present study (around 6.2 °C higher than TN conditions), which were well above their comfort zone. In turn, the high AT recorded under HS conditions explains the dramatic ADG and BW drop in HS birds at 21–35 days of age compared to TN chickens. Although factors related to period differences such photoperiod, relative humidity, among others, might affect performance response, the coincidence with previous reports supports the HS impact observed in our study.

Substantial reduction in feed intake is the response commonly reported for broiler chickens exposed to HS (Geraert et al. 1996; He et al. 2018; Awad et al. 2020; Goel 2021; Kumar et al. 2021). This response is probably aimed at reducing the heat production derived from the feeding and digestion processes (Cervantes et al. 2018), despite an increased expression of gonadotropin-inhibitory hormone, an orexigenic agent (Chowdhury et al. 2012) and other appetite-stimulating hormones such as ghrelin and cholecystokinin, feed intake remained reduced (Song et al. 2012; He et al. 2018). In agreement to those reports, in the present study, from day 21 to 35, HS birds consumed 18% less feed than TN chickens which partially explains the 35% reduction in ADG and 26% depression in feed efficiency of HS birds during the same period.

Discrepancies regarding the effect of probiotics on feed intake and other productive variables in HS birds have been observed. Some authors report general positive effect when supplementing *B. subtilis* or other commercial probiotics or symbiotics that contain this species (Wang et al. 2018; Abdelqader et al. 2020; Du et al. 2023). Other authors (Song et al. 2014; Kazemi et al. 2019), however, reported no positive effects on avian performance. Regardless of the AT, in this study, probiotic supplementation reduced feed intake in birds by around 8%, but improved feed conversion also by 8% from day 21 to 35. These discrepancies may be explained by differences in the environmental conditions, age of the birds, the strains and sources of probiotic utilized, as well as their viability and concentrations in the diet (Jha et al. 2020). The improved feed efficiency observed in the current study could be associated with an enhanced feed digestibility and nutrient retention as previously reported in broilers supplemented with *B. subtilis*-based probiotics (He et al. 2019; Biswas et al. 2023). This suggests a more efficient use of nutrients not only for growth performance, but also to improve gut histomorphology and intestinal barrier. Individual administration of probiotics based on different microorganisms (*Enterococcus faecalis* CV1028*, Bacteroides fragilis* GP1764, and *Ligilactobacillus salivarius* CTC2197) provoked similar improvements in feed conversion (Hussain et al. 2024), suggesting that this response is not exclusive for the probiotic used in the current experiment.

Animals, including chickens, exposed to high AT make some physiological adjustments to conserve homeostasis. Peripheral blood vessels vasodilatation is employed to dissipate body heat, but this response provokes vasoconstriction in internal organs such as the gastrointestinal tract (Siddegowda et al. 2007; Lambert 2009). Reduced blood flow to the small intestine tract reduces the oxygen and nutrient flux to this organ affecting both the epithelial integrity and its barrier function (Yan et al. 2006; Liu et al. 2022). Reduced villus height and elongated crypt depth are associated with the damage observed in the epithelium of animals exposed to high AT (Wu et al. 2018; Liu et al. 2020; Nanto-hara et al. 2020; Zhang et al. 2022). As a consequence the absorptive surface is reduced that, combined with a lower abundance of nutrient transporters (Habashy et al. 2017; Al-zghoul et al. 2019), reduces the availability of nutrients for growth.

In agreement with those reports, HS-S chickens of the present experiment showed reduced villi height, villi width, crypt depth, and VH:CD ratio in jejunum, compared to TN-S chickens. However, the probiotic seemed to help restoring the intestinal epithelia, as the HS-P chickens increased around 30% their villi height, and also villi width and crypt depth increased above of 50% compared to HS-S, although a similar effect was observed in the TN-P. Similar results after probiotic supplementation were reported under both TN (Olnood et al. 2015; Abd El-Hack et al. 2020) and HS conditions (Deng et al. 2012; Song et al. 2014; Li et al. 2020; Salem et al. 2022). Abdelqader et al. (2020) observed that a probiotic based on *B. subtilis*, the same one used in the present study, improved the small intestine epithelial characteristics and the productive performance of HS chickens. Sokale et al. (2019) demonstrated that a probiotic based on *B. subtilis* (DSM 32315) improved the small intestine histological characteristics of chickens challenged with *Clostridium perfringens*, It appears that probiotics positively affect the intestinal morphology of chickens regardless of the AT or the challenge they are exposed to. However, it should be acknowledged that probiotics mode of action, such as the control of gut microbiota populations, colonization by pathogens, and the improvement of performance and intestinal health, may differ depending on the species and even on the strains of same species (Tarradas et al. 2020; Tous et al. 2022).

Heat stress seems to inhibit the proliferation of the small intestine epithelial cells by down-regulating the Wnt/β-catenin signaling pathway, which reduces the proliferation and differentiation of stem cells in the intestinal crypts (Zhou and Zhu 2020). Nevertheless, in the present experiment, the deepest crypts observed in HS-P and TN-P may indicate that the probiotic favored the proliferative capacity of intestinal cells, which in turn served as replacement for shedding cells in the villi.

Tight junction proteins, including claudins, occludins, zonoccludin or TJ proteins, are responsible for maintaining the cell to cell junction integrity and the selective paracellular permeability. Claudins are intercellular proteins located at the apical end of enterocytes, whereas occludins are located further below the claudin junctions, and both claudins and occludins are attached to TJ proteins which interact with actin filaments of the cytoskeleton (Otani and Furuse 2020). Thus, the shortening of villi height in HS birds may be associated with the absence or malfunction of TJP but the results are controversial and vary depending on factors like time, AT, and length of exposition period. Some authors agree that a reduced expression of TJP is linked to increased intestinal epithelial damage, and occurs when birds are exposed to AT above 32 °C for more than 10 h per day during less than 7 days (Zhang et al. 2017; Uerlings et al. 2018; Liu et al. 2022; Du et al. 2023; Peng et al. 2023). In contrast, when AT ranges from 28 to 30 °C and exposition period exceeds 14 days, birds do not show important changes in gene expression of TJP (Goo et al. 2019; Peng et al. 2023), suggesting that birds could get some adaptation to high AT. In agreement, in the present study, AT per se did not affect gene expression of tight junction proteins even when morphology alterations on intestinal epithelium remained.

On the other hand, probiotics appear to increase intestinal villus height and the expression of TJP, which can also be remodeled and redistributed in response to stimuli like nutrients, protein or energy content in the diet (Suzuki 2020). In the present study, compared to HS-S birds, the HS-P birds not only increased villi height by 30% but also had a twofold increment in the expression of claudin-5; probiotic increased 1.6-fold the expression of claudin-5 in TN-P chickens compared to TN-S birds. In agreement with those reports, these results may suggest that the improved small intestine epithelial histomorphology of chickens fed diets supplemented with probiotic is associated with increased expression of tight junction proteins, regardless of the AT they are exposed to. Overexpression of jejunal claudin-5 in HS birds supplemented with the probiotic, could also positively contribute with regulating adequate paracellular permeability at the top of the enterocytes. Thus, assuming claudin-5 ensured the normal permeability at the top, enterocytes may need less occludin located far below claudins to maintain a safe permeability. So, we speculate that this change in tight junction gene expression may be associated with the partial recovery of the jejunal epithelium histo-morphology observed in chickens fed the probiotic supplemented diets.

The results of this study demonstrate that supplementing the diet with a probiotic based on *Bacillus subtilis* contributes to recover the epithelial integrity characteristics, such as intestinal villi height and crypth dept, and to regulate the expression of tight junction proteins in chickens raised under HS conditions, which positively impacts their performance. However, it is necessary to take into account the particular conditions of each farm with regards to the supplemental optimal dose, and choose the specific strains capable to providing the greatest benefit for the birds.

## Data Availability

The data that support the finding of this study are available from the corresponding author upon request.
